# Boosting cancer immunotherapy: drug delivery systems leveraging ferroptosis and immune checkpoint blockade

**DOI:** 10.3389/fimmu.2025.1611299

**Published:** 2025-06-25

**Authors:** Ting Zhang, Fanlin Gu, Wei Lin, Haiyan Shao, Aiguo Jiang, Xingang Guan

**Affiliations:** ^1^ The First People’s Hospital of Wenling (Taizhou University Affiliated Wenling Hospital), School of Medicine, Taizhou University, Taizhou, Zhejiang, China; ^2^ College of Medical Technology, Beihua University, Jilin, Jilin, China

**Keywords:** immune checkpoint inhibitor, ferroptosis, drug delivery, immunotherapy, combination therapy

## Abstract

Immune checkpoint inhibitors (ICIs) have revolutionized cancer treatment, significantly improving outcomes for various malignancies. Despite their clinical success, only a subset of patients benefits from ICIs treatment, underscoring the need for innovative strategies to enhance their therapeutic potential. Ferroptosis, a unique form of programmed cell death driven by iron-dependent lipid peroxidation, has emerged as a promising partner for enhanced immunotherapy. Combining ferroptosis inducers with immune checkpoint blockade has shown promising potential in improving the efficacy of cancer immunotherapy. This study explores the mechanisms of ferroptosis and immune checkpoint inhibitors for synergistic cancer treatment, and reviews recent delivery platforms integrating ferroptosis and immune checkpoint blockade for enhanced therapy.

## 1 Introduction

Over the past decade, immune checkpoint inhibitors (ICIs) have achieved remarkable success in the treatment of various advanced cancers. Antibodies targeting CTLA-4, PD-1/PD-L1, and LAG3, either alone or in combination with other therapies, have significantly improved therapeutic outcomes for cancer patients (1). Despite these advancements, only a subset of patients benefits from current ICI-based strategies. This limited response rate can be attributed to several factors, including poor tumor-associated antigen (TAA) exposure, low MHC molecule expression, and the infiltration of immunosuppressive cells (2, 3). To overcome these limitations, combination therapies have emerged as a promising approach to enhance the antitumor efficacy of ICIs. Combining ICIs with chemotherapy, radiotherapy, anti-angiogenic agents, targeted therapies, or other immunotherapies has demonstrated superior outcomes compared to ICIs alone in treating certain cancers (4, 5). The development of novel combination strategies holds significant potential for improving the effectiveness of ICIs and expanding their therapeutic benefits.

Ferroptosis is a distinct form of regulated cell death characterized by iron-dependent lipid peroxidation and cell membrane damage (6). This process is defined by key features, including iron dependency, glutathione depletion, lipid peroxidation, and unique genetic and biochemical regulation. Unlike apoptosis or necrosis, ferroptosis is driven by the accumulation of reactive oxygen species (ROS), which ultimately leads to oxidative damage of cellular lipids (7). ROS-induced oxidative stress in tumor cells induce immunogenic cell death (ICD) by releasing damage-associated molecular patterns (DAMPs), which promote the activation of dendritic cells and priming T-cell responses (8, 9). Notably, ICIs can also trigger ferroptotic cell death in tumor cells (10). The ICIs and ferroptosis are mutually reinforcing relationships in treating cancers. In mice bearing ovarian or melanoma tumors, activated CD8^+^ T cells induced by ICIs promote enhance lipid peroxidation and tumor cell ferroptosis, and the enhanced ferroptosis in turn potentiates the therapeutic efficacy of immune checkpoint blockade (11).

This review provides a brief overview of the mechanisms underlying the combination of ferroptosis inducers and ICIs to enhance cancer therapy. Furthermore, we explore recent progress in novel drug delivery systems designed to simultaneously induce ferroptosis and amplify immunotherapy. By emphasizing the therapeutic potential of this combination strategy, this review aims to offer valuable insights into innovative approaches for overcoming treatment resistance and improving outcomes in cancer therapy.

## 2 Immune checkpoint inhibitors

Immune checkpoints are a group of membrane receptors expressed on immune cells that play a critical role in negatively regulating immune responses (12). The most well-studied immune checkpoints are cytotoxic T-lymphocyte antigen-4 (CTLA-4), programmed cell death 1 (PD-1), lymphocyte activation gene 3 (LAG-3), T cell immunoglobulin and mucin-containing molecule 3 (TIM-3), and T cell immunoglobulin and ITIM domain (TIGIT) (13). Under normal physiological conditions, these immune checkpoints help maintain immune homeostasis by preventing excessive immune activation, thereby protecting healthy tissues from autoimmune damage. However, during tumorigenesis and cancer progression, tumor cells can exploit this regulatory mechanism by overexpressing specific ligands that bind to immune checkpoints, leading to immune cell dysfunction and suppression of antitumor immunity (14). To date, the U.S. Food and Drug Administration (FDA) has approved ICIs targeting CTLA-4, PD-1/PD-L1, and LAG-3 for the treatment of various advanced cancers (15, 16). These ICIs have demonstrated the ability to induce systemic and durable antitumor immune responses, revolutionizing cancer immunotherapy. Despite the remarkable success of ICIs in oncology, their clinical application faces several key limitations. While ICIs demonstrate significant efficacy in cancers such as melanoma and Hodgkin’s lymphoma, their effectiveness remains limited in many other tumor types (e.g. pancreatic adenocarcinoma, prostate cancer, glioblastoma, ovarian cancer, triple-negative breast cancer, hepatocellular carcinoma) with only a small subset of patients achieving durable responses (10%~40%) (17). Furthermore, immune-related adverse events (irAEs) and the development of drug resistance pose major challenges, further restricting their broader clinical utility (3).

### 2.1 CTLA-4

CTLA-4, also known as CD152, is a transmembrane receptor mainly expressed in activated T cells and regulatory T (Treg) cells (18). Two conditions need to be satisfied for T cell activation: the recognition of antigen by T-cell receptor (TCR) and costimulatory signals between CD28 and ligands (CD80 and CD86). CTLA-4 shares high homology with CD28 and competes for interaction with CD80/86 (19). Due to the higher binding with CD80/86, CTLA-4 inhibits the binding of CD28 with the ligands, resulting in anergy of T cells. CTLA-4 expressed in regulatory T (Treg) cells can exert the suppression effect on T cells and contribute to T cell exhaustion. CTLA-4 blockade can deplete the Treg cells from the tumor microenvironment and facilitate a paradigm shift in immunotherapy (20). Ipilimumab, a human CTLA-4 antibody, was approved by the FDA in 2011 to treat unresectable or metastatic melanoma. Combining ipilimumab with nivolumab was then approved for advanced renal cell carcinoma and metastatic colorectal cancer (21). Tremelimumab, another ICIs targeting CTLA-4, was approved in combination with durvalumab (anti-PD-L1) for treating unresectable hepatocellular carcinoma (HCC) in the USA and Japan as the first-line treatment of adults (22).

### 2.2 PD-L1/PD-1

PD-1 (also named PDCD1 and CD279) is a checkpoint receptor expressed in T cells, B cells, natural killer cells, and some myeloid cell populations. PD-1 has two ligands programmed death ligand 1 (PD-L1) and programmed death ligand 2 (PD-L2) (23). PD-L1 is expressed in many types of cells including tumor cells, immune cells, epithelial cells, and endothelial cells, while PD-L2 is only detected in antigen-presenting cells (APCs). The interaction of PD-1 with its ligand is involved in T cell activation, proliferation, and cytotoxic cytokine secretion (24). In tumor microenvironment, the PD-L1/PD-1 axis is responsible for the maintenance of immune tolerance and the immune suppressive environment. Disrupting the PD-L1/PD-1 axis can restore the normal function of T cells and elicit an antitumor immune response (25, 26). Monoclonal antibodies targeting this axis, including nivolumab, pembrolizumab, cemiplimab, atezolizumab, avelumab, and durvalumab have been approved by FDA to treat advanced melanoma and other cancers. Nivolumab was the first approved ICIs targeting PD-1 in 2014 for unresectable and metastatic melanoma. Later, the FDA approved its use in treating squamous non-small cell lung cancer (NSCLC), small-cell lung cancer, advanced renal cell cancer, Hodgkin’s lymphoma, metastatic squamous cell cancer of head and neck, metastatic urothelial cancer, metastatic colorectal cancer, HCC (21). Despite their remarkable success in treating certain advanced cancers, ICIs targeting the PD-1/PD-L1 axis exhibit several limitations in clinical practice. First, heterogeneous patient responses limit their therapeutic efficacy, with only a subset of individuals achieving durable benefits. Second, ICIs frequently induce irAEs, including gastrointestinal toxicity (e.g., colitis), dermatologic reactions (e.g., rash, vitiligo), pneumonitis, endocrinopathies (e.g., hypothyroidism), and hepatic or renal dysfunction. Third, the development of primary or acquired resistance remains a major barrier to expanding their clinical utility (17).

### 2.3 LAG-3

In 2022, relatlimab, a monoclonal antibody targeting LAG-3, in combination with Nivolumab, was approved for the treatment of metastatic melanoma, making LAG3 the third clinically utilized immune checkpoint after CTLA-4 and PD-1 (15). LAG-3 is primarily expressed in activated CD4^+^ and CD8^+^ T cells to prevent autoimmunity. Sustainable antigen exposure promotes LAG-3 expression as well as other inhibitory receptors on T cells, leading to a state of T cell exhaustion (27). LAG-3 is also constitutively expressed in regulatory T cells. LAG-3 has four ligands, including major histocompatibility complex class II (MHC II), galectin-3 (Gal-3), fibrinogen-like protein 1 (FGL1), and liver sinusoidal endothelial cell lectin (LSECtin). LAG-3 inhibits T cell proliferation and cytokine secretion through the selective binding with MHCII. Clinical studies have demonstrated the high expression of LAG-3 in many tumors including NSCLC, melanoma, and ovarian cancer. LAG-3 expression is positively correlated with poor prognosis and disease-free survival (28). Relatlimab can bind to the LAG-3 receptor and prevent its interaction with MHCII ligands, restoring T cell’s mediated immune attack against tumor cells.

## 3 Ferroptosis

### 3.1 Morphology and biochemical feature of ferroptosis

Ferroptosis, a type of iron-dependent regulated cell death, is characterized by uncontrolled lipid peroxidation and induced plasma membrane rupture (6). Ferroptotic cell death is different from reported cell death, such as apoptosis, necrosis, and autophagy. The morphology features of ferroptosis include the ruptured plasma membrane, cytoplasmic organelles swelling, and chromatin condensation. (29). Iron accumulation and lipid peroxidation present two standard biochemical features of ferroptosis. Ferroptosis inducer or glutathione peroxidase 4 (GPX4) inhibitors can contribute to the increased intracellular iron accumulation, which facilitates ROS production through the Fenton reaction and promotes the lipid peroxidation of polyunsaturated fatty acids (PUFA). The aggravated lipid peroxidation produced excessive toxic products, which finally smashed the cell membrane (6).

Iron, an essential element for cellular metabolism, plays a critical role in free radical generation and lipid peroxidation (30). Extracellular Fe³^+^ is internalized via transferrin receptors and subsequently reduced to Fe²^+^, which enters the labile iron pool (LIP). Due to its high reactivity, Fe²^+^ catalyzes the Fenton reaction, generating hydroxyl radicals that directly oxidize PUFAs in the plasma membrane. This process produces excessive lipid ROS, ultimately triggering cell death (10). Iron homeostasis is tightly linked to ferroptosis: iron accumulation promotes ferroptosis, whereas enhanced iron export suppresses it (31, 32). Key regulators of iron uptake and export influence ferroptotic susceptibility (33). Gene interventions such as iron chelation or iron overload can inhibit ferroptotic cell death (34, 35). For example, NRF2 silencing or HSPB1 knockdown leads to iron accumulation in tumor cells, sensitizing them to ferroptosis (36).

Lipid peroxidation is a hallmark of ferroptosis (37). In the presence of Fe²^+^, ROS and lipoxygenase oxidize PUFA in cell membranes, generating toxic lipid peroxidation products that ultimately cause membrane rupture and cell death (38). The role of lipid peroxides in ferroptosis varies across tumor types. For instance, in gastric cancer, lipid peroxides trigger ferroptosis via the GPX4/SREBP-1α pathway, suppressing tumor cell growth (39). Conversely, in colon cancer, reduced intracellular lipid peroxidation protects cells from ferroptosis and promotes liver metastasis via SLC7A11 (40).

### 3.2 Ferroptosis improves the efficacy of chemotherapy and radiotherapy

The dysregulated ferroptosis is associated with cancer, inflammation, neurodegeneration, and other diseases (41). The combination use of chemotherapeutic drugs (e.g. oxaliplatin, cisplatin, gemcitabine, and 5-fluorouracil) and ferroptosis inducers has addressed drug resistance and improved the therapeutic outcomes of chemotherapy (42). For example, erastin, a ferroptosis inducer, can sensitize cancer cells to cisplatin treatment, enhancing the cytotoxicity of chemotherapeutic drugs (43, 44). An immunosuppressant drug sulfasalazine impedes the cellular uptake of cysteine and contributes to ferroptosis in cancer cells (45). Radiotherapy can contribute to excessive ROS production and promote lipid peroxidation in lipid bilayers, resulting the ferroptotic cell death (46). Notably, the combination of ferroptosis inducers and radiotherapy can inhibit more tumor growth than radiotherapy alone (47). Many studies have validated the sensitizing effect of ferroptosis inducers in enhancing the efficacy of radiotherapy (47).

### 3.3 Ferroptosis and immunotherapy

**Figure 1:**
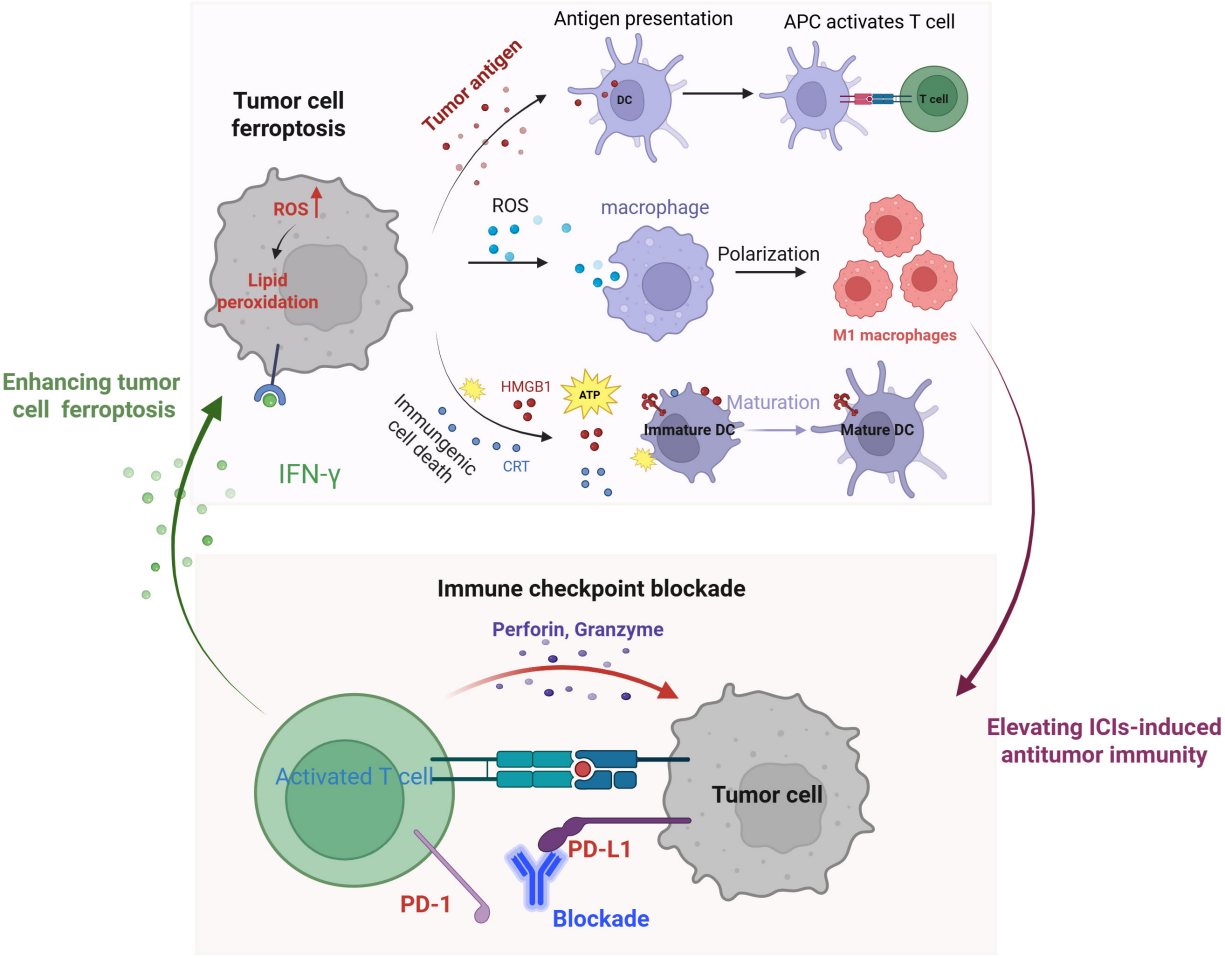
Schematic illustration of the mechanisms of tumor cell ferroptosis for potentiated immunotherapy and immune checkpoint blockade for enhanced ferroptosis.

The clinical application of immune checkpoint inhibitors (ICIs) is significantly limited by their low response rates, adverse effects, and drug resistance. Ferroptosis represents a promising strategy to enhance immunotherapy efficacy (48) (**Figure 1**). Emerging evidence suggests that ferroptosis effectively kills tumor cells while promoting the release of TAAs, thereby stimulating antitumor immunity (49). Furthermore, ferroptosis inducers can trigger ICD in tumor cells, leading to the release of high-mobility group box 1 (HMGB1) and ATP into the tumor microenvironment, as well as the plasma membrane translocation of calreticulin (CRT) (50, 51). These aberrant releases and translocations serve as “find-me” signals, recruiting immune cells to the tumor site. Additionally, ferroptotic cancer cells in early stages facilitate the maturation of bone marrow-derived dendritic cells (BMDCs), inducing a vaccination-like effect in immunocompetent mice (52). Notably, ROS generated during ferroptosis drive the repolarization of tumor-associated macrophages (TAMs) from the immunosuppressive M2 phenotype to the antitumor M1 phenotype (53). Together, these findings underscore the pivotal role of ferroptosis in activating tumor antigen-specific immune responses (54).

Ferroptosis significantly enhances the efficacy of ICIs-mediated immunotherapy, while ICIs, in turn, potentiate tumor cell ferroptosis (48). This mutually reinforcing relationship positions ferroptosis and ICIs as an ideal combination for cancer treatment. Mechanistically, activated CD8^+^ T cells release interferon-gamma (IFN-γ), which suppresses cystine uptake in tumor cells, promoting lipid peroxidation and ferroptosis (11). Additionally, IFN-γ secreted by T cells in conjunction with arachidonic acid (AA)—induces immunogenic tumor ferroptosis, further amplifying immune checkpoint blockade-driven antitumor immunity (55, 56). Collectively, these findings highlight the synergistic interplay between ferroptosis and immunotherapy, offering a promising strategy for enhanced cancer treatment.

## 4 Drug delivery systems for ferroptosis and ICIs therapy

Given the great potential of combining strategy in enhanced therapy, the combination of ferroptosis and immunotherapy represents a promising treatment for improved efficacy. Due to the non-specific distribution of ferroptosis inducers, efficient delivery of ferroptosis inducers and ICIs remains a significant challenge (57–59). Drug delivery systems can improve drug-loading efficiency and bioavailability, and enhance tumor distribution and antitumor effects (60, 61).

**Figure 2:**
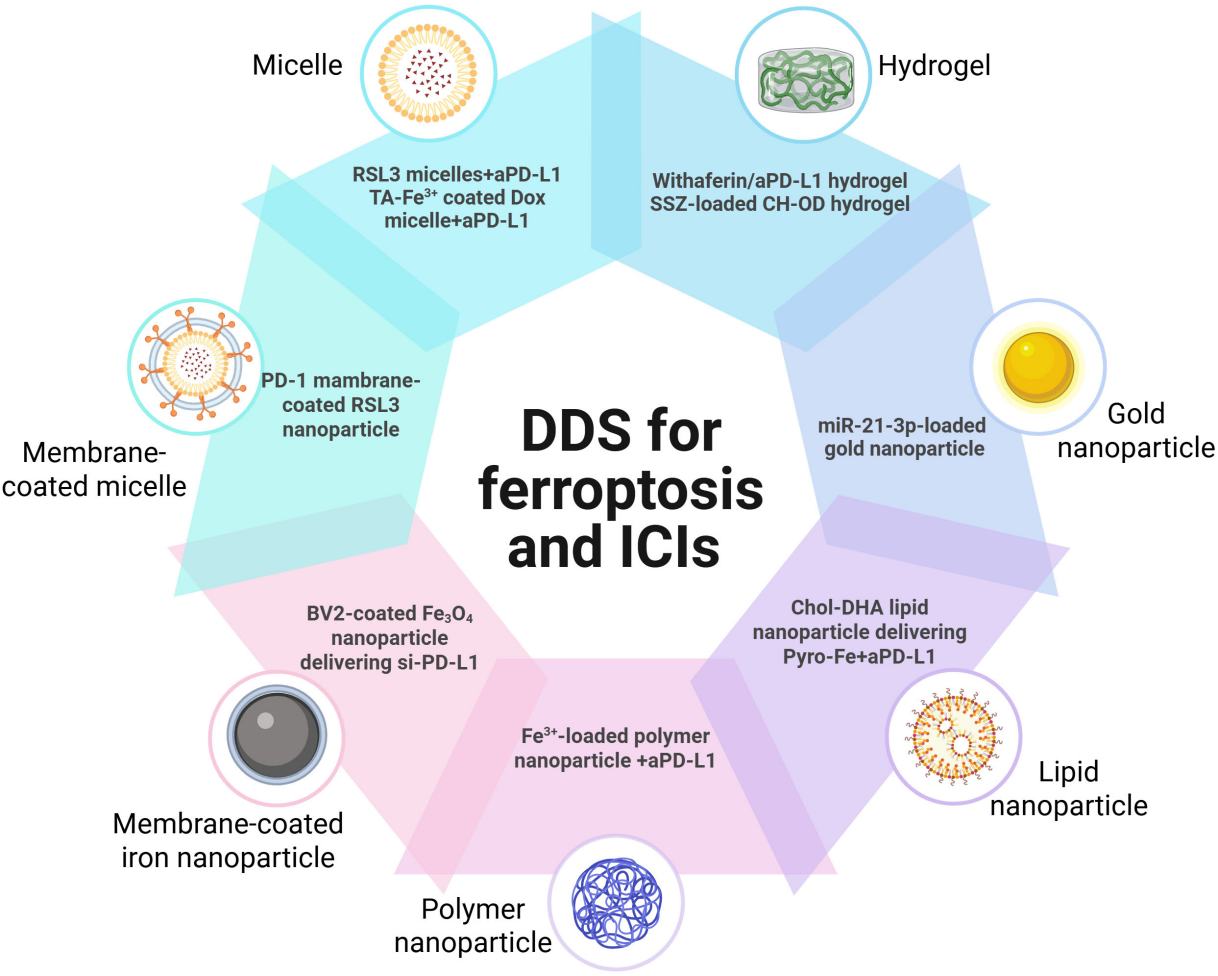
Drug delivery systems for ferroptosis and ICIs therapy.

Owing to the synergist effect of combining ferroptosis and ICIs in antitumor treatment, many delivery platforms have been developed for ferroptosis-based combination therapy (50, 62) (**Table 1**). Song developed an acid-activate nanoparticle using an ionizable block copolymer and acid-liable phenylboronate ester (PBE) for delivery of glutathione peroxidase 4 inhibitor RSL-3. This nanoformulation exerts acid-activatable photodynamic therapy and sensitizes the tumor cells to RSL-3-inducible ferroptosis. Combining this nanoparticle and anti-PD-L1 significantly inhibits tumor growth and lung metastasis (63). Mu prepared PD-1 membrane-coated RSL3 nanoparticles for combination cancer therapy. The PD-1 receptors-decorated on the surface could disrupt the PD-1/PD-L1 axis and trigger antitumor immunity in breast cancer. In addition, RSL3-loaded nanoparticles promote tumor cell ferroptosis through GPX4 inhibition and elevate antitumor immune response (62). Jeong fabricated a tannic acid (TA)-Fe^3+^-coated doxorubicin (DOX) nanoparticle for ferroptosis/apoptosis-based combination therapy. The combination of this nanoparticle and anti-PD-L1 substantially delayed tumor growth and improved antitumor immune response with increased CD4^+^ and CD8^+^ T cell infiltration and decreased numbers of Treg (64). Cheng designed a sodium alginate-based hydrogel for local delivery of Withaferin prodrugs and antiPD-L1. The loaded Withaferin prodrugs induced tumor cell ferroptosis and the release of tumor antigens, synergistically improving antiPD-L1-elicited antitumor immunity (65). Cai developed a hypoxia-responsive nanoparticle using coordinating ferric (Fe^3+^), mitoxantrone (MTO), and sulfasalazine for ferroptosis-enhanced immunotherapy. The nanoparticles enable stimuli-responsive delivery of iron and MTO, synergistically enhancing ROS generation. Combining with sulfasalazine-induced lipid peroxidation, this system potently amplifies tumor cell ferroptosis. Furthermore, these nanoparticles demonstrate remarkable synergy with anti-PD-L1 therapy, significantly improving immunotherapeutic efficacy through enhanced ferroptosis-immunity crosstalk. (67). Liu prepared microglial membrane-coated Fe_3_O_4_ nanoparticles to deliver siRNA-PD-L1 for treating glioblastoma. The Fe_3_O_4_ nanoparticles provided sufficient Fe^2+^ for ferroptosis in drug-resistant GBM cells, and siRNA-mediated PD-L1 downregulation promoted DC maturation and T-cell activation (70). Ding fabricated a neutrophil-targeted polymer nanocarrier for co-delivering siPD-L1 and Fe_3_O_4_. This nanoformulation exerted sono-activatable combination immunotherapy, leading to inhibited glioma tumor growth and improved mouse survival (72). These engineered drug delivery systems demonstrated high loading efficiency and tumor-specific release of ferroptosis inducers. By robustly inducing tumor cell ferroptosis and subsequently activating antitumor immunity, these systems significantly potentiated immunotherapy responses. These stimuli-responsive and multifunctional delivery systems demonstrates significant potential to enhance the therapeutic efficacy of combined ferroptosis and immunotherapy approaches (**Figure 2**).

**Table 1:** Representative delivery platforms integrating ferroptosis and immune checkpoint blockade for cancer immunotherapy.

Delivery platforms	Ferroptosis inducers	Blockade agents	Immunotherapy functions	Ref.
PEG-PDPA nanoparticles	RSL3	Anti-PD-L1	Promoted DC maturation, IFN-γ^+^CD8^+^ T cell infiltration, decreased the frequency of Tregs.	(63)
DSPE-PEG micelles	RSL3	PD-1 protein	Promoted DC maturation, CD8+ T cell infiltration and Treg depletion	(62)
DSPE-PEG micelles	tannic acid (TA)-Fe^3+^	Anti-PD-L1	Activating CD4^+^ and CD8^+^ T cells and decreasing regulatory T cells (Treg)	(64)
alginate-calcium hydrogel	Withaferin A	Anti-PD-1	Increasing the infiltration mature DCs, M1 macrophages, CD4^+^ and CD8^+^ T cells	(65)
CH-OD hydrogel	sulfasalazine	anti- PD-1	Promoted DCs maturation, activation of M1 type TAMs, cytotoxic T lymphocytes (CTLs) and Th1 cells	(66)
PEG-Azo-dopamine	Fe^3+^, mitoxantrone, and sulfasalazine	Anti-PD-L1	promote dendritic cells maturation, polarization to M1 macrophages, and T cells activation,	(67)
core-shell nanoparticles	pyropheophorbide-iron (Pyro-Fe)	Anti-PD-L1	Increasing the infiltration of CD4^+^ T cells, CD8^+^ T cells, NK cells, and B cells	(68)
gold nanoparticles	miR-21-3p	anti-PD-1	Increased infiltration of CD8^+^ T cells and F4/80^+^CD11b^+^ macrophages	(69)
Fe_3_O_4_ nanoparticles	Fe_3_O_4_	PD-L1 siRNA	Increased the ratio between effector T cells and regulatory T cells	(70)
PEG-PLGA nanoparticles	Fe/FeO	anti-PD-1	Promote the infiltration of CD4 and CD8-positive T cells	(71)
DSPE-PEG micelles	Fe_3_O_4_	PD-L1 siRNA	Increased infiltration of matured DCs, CD4^+^ T cells, and CD8^+^ T cells. Decreased Treg.	(72)
PNPs nanoparticles	cGAMP, Fe^3+^	anti-PD-L1	increased the infiltration rate of CD4^+^ and CD8^+^ T cells	(73)

## 5 Challenges and perspectives

Immune checkpoint blockade could disrupt the immunosuppressive signals and revive the exhausted immune systems for systematic and durable immunotherapy (25, 74). However, the clinical application is hindered by the poor exposure of tumor-associated antigens, infiltration of suppressive immune cells (e.g. Treg, MDSC), and others (17). Tumor cell ferroptosis can increase the release of tumor antigens and activate antitumor immune response, facilitating the immune checkpoint blockade for immunotherapy (50, 75). Ferroptosis/immunotherapy-based combination presents an effective strategy to overcome the limitations of current ICIs.

Advanced drug delivery systems enable tumor-selective release of ferroptosis inducers and immune checkpoint inhibitors, significantly enhancing therapeutic efficacy. However, several critical factors must be addressed for optimal ferroptosis/ICI combination therapy. First, emerging evidence suggests that ferroptosis may impair tumor antigen presentation and adaptive immune responses in certain contexts, potentially limiting immunotherapy efficacy (76). Therefore, careful selection of appropriate ferroptosis inducers is crucial for achieving optimal immunotherapeutic outcomes. Second, given the potential toxicity of ferroptosis inducers to healthy tissues and ICIs-caused irAEs (6, 77), tumor-targeted delivery systems are essential to maximize synergistic antitumor effects while minimizing systemic toxicity. Third, given the enhanced antitumor immune response triggered by this combination, co-delivery platforms must carefully balance therapeutic efficacy with potential immune overactivation. This requires precise optimization of both ferroptosis inducer dosing and the ferroptosis inducer/ICI ratio to maximize synergy while minimizing adverse effects. Furthermore, future studies will elucidate the detailed molecular mechanisms underlying (1) how ferroptosis inducers modulate immune responses, and (2) how immune checkpoint blockade influences tumor cell susceptibility to ferroptosis. A comprehensive understanding of these underlying mechanisms will be critical for the rational design of safe and efficient delivery systems with optimized therapeutic efficacy.
